# Young People’s Voices and Science for Overcoming Toxic Relationships Represented in *Sex Education*

**DOI:** 10.3390/ijerph19063316

**Published:** 2022-03-11

**Authors:** Beatriz Villarejo-Carballido, Cristina M. Pulido, Harkaitz Zubiri-Esnaola, Esther Oliver

**Affiliations:** 1 Department of Journalism and Communication Studies, Autonomous University of Barcelona, 08193 Bellaterra, Spain; beatriz.villarejo@uab.cat (B.V.-C.); cristina.pulido@uab.cat (C.M.P.); 2 Department of Language and Literature Didactics, University of the Basque Country UPV/EHU, 20018 San Sebastian, Spain; harkaitz.zubiri@ehu.eus; 3 Department of Sociology, University of Barcelona, 08034 Barcelona, Spain

**Keywords:** fiction series, toxic relationship, young, socialisation, dialogic interaction, impact

## Abstract

The scientific literature has presented evidence of how fiction series impact the socialisation of young people’s relationships. However, there is a gap in the evidence on how dialogic interactions overcome the negative impact of the fiction series on the socialisation of toxic relationships. This research analyses dialogic interactions based on scientific evidence related to toxic relationships that contribute to overcoming this type of relationship. First, we developed a communicative content analysis of eight episodes of one of the most-watched fiction series by young people, *Sex Education*. After that, we conducted four communicative interviews with the young audience to collect their voices about the impact of these toxic relationships represented in this fiction series in their daily conversations. The results indicate that in such cases, there is a need to promote dialogic interactions about this fiction series, focusing the dialogue on which type of masculinity develops a toxic relationship and which, on the contrary, promotes healthy relationships.

## 1. Introduction

Since the mid-twentieth century, several types of research have shown how audiovisual products can generate effects on the audience and their own vision of reality, especially on the youngest [1,2,3,4]. One of these is the research *Cultural Indicators* [5], which measured violence on television and its effects on society. More than 4000 scenes from different programmes and measurements of violent scenes were used as the sample. The research team found that people who are more exposed to the screen are more influenced by media content than those who are not, which they termed “mainstreaming” [5]. They further stated that if the person’s lived experiences are in line with what is broadcast, this influences the viewer twice as much, resulting in an increase in the degree of cultivation [5].

Today more than twice as many young people watch videos on a daily basis [6], making them the main consumers of audiovisual products. The emergence of digital platforms has changed young people’s viewing habits; they decide what to watch and when [7,8]. Young people watch television series through these platforms, which provide the young person with the entertainment function, the informative function, and various social functions [9]. In relation to the way young people consume these products, several studies show that the contents of the series consumed by boys and girls are different. While girls are more likely to prefer to watch more romance and drama, boys prefer to watch action and humour [10,11] and, as mentioned earlier, the content consumed influences the viewer.

### 1.1. Impact of Fiction Series on the Socialisation of Young People’s Affective-Sexual Relationships

In fiction series, there is a proliferation of images and language related to the characters. Many of these can provoke emotions and influence the values and behaviours of the audience [12,13,14].

Thus, it is found that youth series aimed at a young audience have significant value, both for television production and the reception of the audience, who are at a time of identity construction [15]. Ward and Rivadeneyra [16], who examined 314 young students, found a correlation between the amount of audiovisuals viewed and participants’ sexual attitudes, expectations, and behaviours.

The audiovisual narrative is the backbone of the story, which changes according to the audiovisual genre and the target audience niche [17]. Love relationships are one of the most common themes in audiovisual storytelling, mainly because they reign in the world of dreams and the desires of the audience [18]. Wexman [19] points out that the power of the discourse produced through the representation of love relationships impacts romance styles in society. Ward [20], who researched 259 young people, stated that there was a correlation between what they consumed and their sexual attitudes and assumptions.

Sexuality is another key theme in youth series, as it is key in the transition from childhood to adulthood. Sexuality is represented today with more characters of different sexual orientations and genders than in the previous era. [21]. An example appears in the series for teenagers *Sex Education*, which, according to Marchini [22], represents a sexuality that has been silenced until now by moral and religious dogmas. Dudek, Woode, and Green [23], who also analysed the ways in which young people are represented as producers and consumers of pornographic/erotic narratives in this same series, found that this content can provide sexual information and knowledge.

Affective-sexual relationships in fictional series are often associated with conflicts [15], which can take various forms of physical and/or psychological violence. Berridge [24], who explored narratives of sexual violence in U.S. programmes between 1990 and 2008, found that many depictions of sexual violence appear within the sexual culture.

Bleakley, Jamieson, and Romer [25] analysed the highest-grossing films between 1950 and 2006, a total of 855 products. The results were that male characters are more likely to be portrayed as violent, while female characters are more likely to participate in and be depicted in sexually explicit scenes. The authors concluded that violence and explicit sex has increased in both male and female characters. Several fiction series have appeared depicting violence in affective-sexual relationships, such as *Buffy the Vampire Slayer* (1997–2003), where sexual and mutant relationships abound, where hatred becomes love and violence becomes erotic entanglements [26]; *Los Protegidos* (2010–2012), where one of the affective-sexual relationships between two adolescents is linked to abuse [27]; *Breaking Bad* (2008–2013), in which the relationship between Walter White, the protagonist of the series, includes the protagonist trying to isolate, degrade, exploit, frighten and control his wife Skyler. These tactics are well documented in studies focused on abuse. Thus, the author points out that the lack of emphasis on instances of coercive control by writers and directors makes her partly responsible for contributing to a culture of misogyny. Moreover, accusations of victim-blaming by fans of the series towards Skyler are evident in numerous online blogs, fan forums, and social media platforms [28]. In the same vein, Iftene [29], in his study on the series *American Horror Story* (2011–2021) found that in 2011, the producers added familiar horror film subgenres to the rewrite, using genre strategies in five seasons. Producers made aggressive use of para-cinematic techniques to construct a cult product out of hyper-sexualized horror imagery and narratives.

Therefore, these fiction series that relate affective-sexual relationships with violence can cultivate in viewers an understanding and tolerance of violence in such relationships.

### 1.2. Toxic and Health Relationships

In addition to the influence that media content generates in the audience, it should be considered as well that affective-sexual relationships have a direct impact on people’s health, both emotionally and physically. Depending on the type of affective-sexual relationship that is established, it can have a positive or negative impact on health.

Domination and discrimination are two components that indicate the poor quality of a relationship, which is called a toxic relationship [30] and produced in heterosexual and/or homosexual relationships [31,32,33]. These elements of power and dominance appear in Dominant Traditional Masculinity (DTM) [34,35], masculinity that can be socialised to suppress emotions in order to maintain dominance of women [36]. Likewise, Connell [37] notes that these types of masculinity are always violent.

In contrast, healthy relationships experience a higher level of relational satisfaction and positive affect [38]. These types of relationships are fostered by men who belong to the model called the New Alternative Masculinity (NAM), that combines good values and attraction [34]. For this reason, they seek affective-sexual relationships based on desire and love, thus distancing themselves from people with non-egalitarian and/or violent values. This type of masculinity is characterised by self-confidence, bravery, and courage [34]. Furthermore, this masculinity shows rejection of negative attitudes such as sexism, racism, and double standards [39,40]. Therefore, this masculinity publicly rejects non-egalitarian attitudes.

These influences on health, in addition to the emotional, can have physical effects. Recent research by Chuang [41] found that romantic relationships are associated with increased gut microbiota diversity and other health benefits. However, when these romantic relationships go through “heartbreak” or “post-relationship grief”, universal life stress occurs that affects the microbiota. Other research highlights that poor-quality affective-sexual relationships can lead to depression [42], anxiety [43], fear [44], anguish [45], memory disturbances [46], emotional disorders [47], environmentally-sensitive physiological impairment (e.g., of central nervous, endocrine, and immune systems) [48], different somatic symptoms [49], and even an increase in the tendency to commit suicide in some people [50]. In addition, there is increased dissatisfaction with the relationship [51].

### 1.3. Science-Based Dialogic Interaction about Health in Relationships

Sociological studies show how a dialogical turn has taken place throughout society in recent decades [52], which affects human relationships. This dialogue is key to fostering societal transformation [53].

This dialogue can also contribute to changes in and improvement of affective-sexual desires and attraction. This is demonstrated by two studies related to socialization in affective-sexual relationships. The first study deals with the social impact on psychology in the field of gender violence in adolescence. Conducted by Racionero and other authors [54], a change was observed in the behaviour of girls who decided to talk about health and toxic relationships. They conducted seven interventions with 15–16-year-old girls in the framework of the research programme on preventive socialisation of gender violence. Specifically, spaces for dialogue were established between the research team and the research participants to support the free reconstruction of mental and affective models of attraction through critical analysis of the dominant coercive discourse. Thus, the young women were able to better understand their own and others’ affective-sexual thinking, emotions, and behaviours in favour of the rejection of violence and the dialogue supported the modification of adolescent girls’ sexual preferences for different types of men. At the end of the research, they observed that some participants used the knowledge gained in the project to help their friends and communities reflect on patterns of coercive sexual attraction, the quality of their intimate relationships, and the different effects of sexual violence and toxic relationships on health. In addition, some of the girls decided to end their toxic relationships after the interventions.

The second study refers to the MEMO4LOVE research, through the publication from Padrós-Cuxart, Molina-Roldán, Gismero, and Tellado [46]. They conducted a questionnaire (*n* = 141) to find adolescents’ peer interactions that promote healthy or toxic affective-sexual relationships and conducted five communicative focus groups with boys and girls. They found that the impact of sharing evidence of the adverse effects of toxic relationships with violent masculinities on health with adolescents produced a transformation of the peer group. In particular, non-violent boys gained self-confidence and girls reoriented or reinforced their attraction to non-violent boys.

Therefore, dialogue spaces that deal with love or affective-sexual relationships can generate a change in choices and tastes that can enhance toxicity-free relationships. Despite all the scientific contributions made to date, it is still unknown how science-based dialogic interactions overcome the negative impact of fiction series on the socialisation of toxic relationships. The question that this research seeks to clarify is whether the interactions with young consumers of this audiovisual content, through the use of scientific evidence in the discussion, can help them to avoid the mental and physical health effects caused by toxic relationships. Knowing whether this type of interaction produces a change in young people will help to eradicate toxic relationships and avoid the health problems caused. To do this, we used for our research the series *Sex Education*, one of the most-watched series of adolescents today. In order to identify how dialogic interactions on fiction series help to identify toxic relationships that cause health consequences, we started with analysis of one of the stories represented in *Sex Education*, where the boy identified as having Dominant Traditional Masculinity treats badly both a girl and a boy with whom he has or has had affective-sexual relationships and the consequences of them on health of both the girl and the boy.

## 2. Materials and Methods

Communicative Methodology [55,56] has been applied in this research, as it includes the voices of the people investigated throughout the process on topics such as communication and health, with the aim of fostering a transformative impact on the research subjects themselves [57].

**Figure 1:**
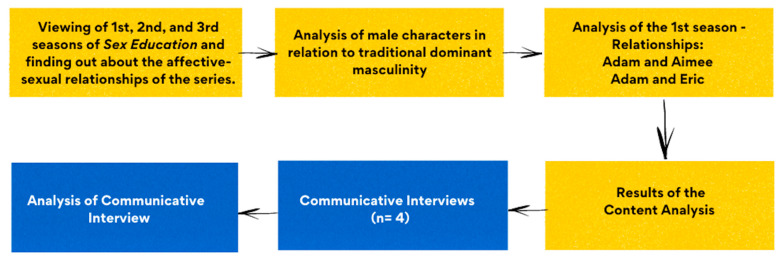
Flowchart of the Data Collection and Analysis process.

We started by watching seasons 1, 2, and 3 of the series *Sex Education* in order to know the characters and their relationships, then we started the review by carrying out a communicative content analysis of the first season of the fictional series *Sex Education*, broadcast by Netflix. Subsequently, communicative focus groups with a communicative orientation were conducted with young girls and boys in relation to two toxic relationships that appear in the eight episodes of the series. Figure 1 shows the data collection process established for this research.

### 2.1. Communicative Content Analysis Data Collection

For this study, we first conducted a communicative content analysis [58] of the first eight episodes of the series. To analyse them, the research team previously watched the three seasons of the fiction series (24 episodes) in order to find out about the affective-sexual relationships that appear throughout the series. Once the affective-sexual relationships were identified, we proceeded to analyze the male characters who meet the characteristics of traditional masculinity, that is, who inflict physical, mental, or sexual harm or suffering, threats to commit such acts, coercion, and other forms of deprivation of liberty on the person with whom he establishes the affective-sexual relationship. In this case, the character of Adam, who assaults and intimidates other people and has very few academic prospects, represents toxic masculinity. Adam is a character who, in the second season, thinks he may be bisexual and has two affective-sexual relationships. The first relationship is heterosexual, with Aimee, a popular high school girl. The second relationship is a homosexual relationship with Eric, a boy who openly says he is homosexual and who is harassed by Adam.

### 2.2. Analysis of Sex Education

The chapters were analyzed in November 2021 with the MAXQDA program, specifically the scenes where the two affective-sexual relationships established by the three characters mentioned in the previous point were visualised or discussed. A researcher, who was trained in this type of analysis, made three visualisations for each of the chapters and categorised those scenes that produced consequences on health (see Table 1). The subcategories were Mental Health and Physical Health. This category and subcategory were previously subtracted from the scientific literature. Once analysed, they were supervised by another member of the research team and discussed with the research team. As a result of the dialogue between the researchers, the final content analysis was confirmed and the first results were obtained.

### 2.3. Communicative Interview Data Collection

Immediately after obtaining the results of the Communicative Content Analysis, we selected two girls and two boys aged 16–24 who consume series on the online platform to whom we asked questions such as “Do you think that the health consequences of being with a partner who is a Dominant Traditional Masculinity are adequately reflected in fiction series?” In addition, four Communicative Interviews were conducted to see what discourse they had about the health consequences of toxic relationships. Table 2 shows the profiles of the interviewees through the Communicative Interview.

The young people and the parents of the minor were previously informed and signed or orally reported ethical consent, which was previously passed by the Ethics Commission of the CREA (Community of Research on Excellent for All) and obtained the reference number 20211228. In this way, this research adheres to international ethical standards related to data collection. The data from the Communicative Interviews were appropriately coded and anonymised.

In these interviews, a researcher discussed with the young people the results obtained from the scientific literature and the content analysis in this study. Based on this information, the interviewees watched two videos with scenes from the first season of the series, specifically the relationship between Adam and Aimee first and between Adam and Eric second. Interviewees reflected on the two relationships and their impact on health. The interviews were recorded and a verbatim transcript of the young people’s contributions was made.

### 2.4. Communicative Interviews Analysis

The Communicative Interviews were then analysed in the previously mentioned research programme. In this case, the categories have emerged from the theoretical contributions made with the object of study and have included the interpretation orientation of the analysis of the applied methodology [55]. Therefore, the analysis of the contributions of the young people interviewed focused on the following: on one hand, on the verbal language that does not relate DTM to negative health consequences, referred to as exclusion, and on the other hand, on the verbal language that identifies the relationship displaying DTM with negative health consequences or healthy relationships with positive health consequences, referred to as transformative (see Table 3).

## 3. Results

The dialogic interactions with scientific contributions and the experiences lived by the group of young people have favoured the contribution to overcoming and detecting toxic affective-sexual relationships that cause negative effects on health.

The results obtained by each of the research tools are shown below.

### 3.1. Results of the Content Analysis

**Figure 2:**
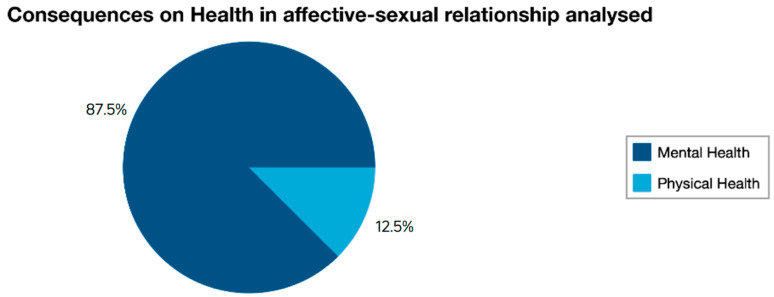
Results concerning the affective-sexual relationships analysed.

In the first season of the fiction series, there are 28 scenes in which the health consequences of having a toxic affective-sexual relationship displaying traditional dominant masculinity appear. Of these scenes, 21 are linked to Adam’s relationship with Eric, while seven are related to Adam’s relationship with Aimee (see Figure 2).

Adam’s affective-sexual relationship with Aimee is a stable relationship, but lasts exactly until the second episode of the season. However, after ending their relationship, Adam starts beating the boys who are related to Aimee. First, in the second episode, he beats up a guy she was flirting with at a party. Then, in the seventh episode, he hits Aimee’s then-boyfriend. The relationship is not a healthy one. Specifically, Aimee appears in seven scenes where we see the negative consequences for her mental health. The protagonist has sexual insecurities, feels uncomfortable with Adam’s insistence on wanting to be invited to the party she organises, and suffers stress and anxiety when she learns that her ex-boyfriend violently entered her house without her consent and when he starts hitting the boys with whom Aimee has some kind of affective-sexual relationship. Thus, although the protagonist of this relationship has not suffered physical violence, the two boys with whom she had some kind of affective-sexual relationship have suffered it.

**Figure 3:**
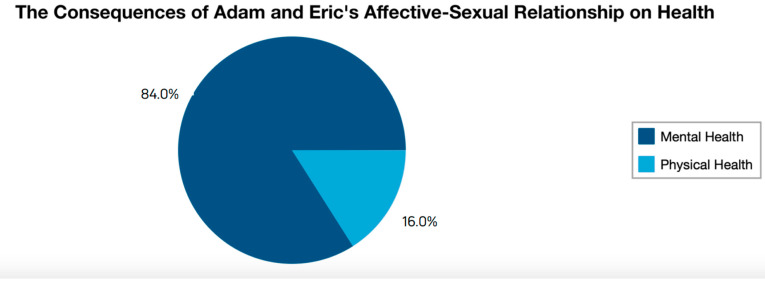
Main results on the negative health consequences of Adam and Eric’s affective-sexual relationships.

Adam’s affective-sexual relationship with Eric is a toxic relationship from the first chapter to the last episode analysed (see Figure 3). Eric is a victim of harassment by Adam, both mental and physical. This relationship causes the victim to live in situations of continuous violence, specifically in 21 scenes in which the consequences on health are seen, 21 to mental health, among which there are four scenes in which there are also consequences on physical health.

The scenes where the victim is seen to have mental health effects are related to feelings of sadness or discouragement, confused thoughts about Adam’s behaviours, excessive worries or fears about any event that links the two of them, and occasional excessive anger with the people around them. His mental situation sometimes pushes him to the edge, especially when problems with his best friend or issues of his sexual identity get mixed up. Within the scenes of consequences experienced by the protagonist of this relationship, the mental and physical consequences are mixed in four scenes. Situations of physical violence are related to physical health effects such as battering and also to the mental health consequences discussed earlier in this paragraph. It should be noted that one of the most violent scenes, both physically and mentally, ended with a sexual intercourse in which the victim felt pleasure.

### 3.2. Transformation through Science-Based Dialogic Interaction

The interviewees state that they do not usually talk about the affective-sexual relationships depicted in the series with their friends. However, they may do so at some point with a close relative. However, they point out that it is not an act that is carried out frequently, and if it is, it is not with scientific information about affective-sexual relationships and the effects on health.

Two of the people interviewed who had followed the series said that the first time they watched the series, they liked it, mainly because it talked about sexual topics that are taboo, such as sexual practices or diseases.

“The first season, I liked it a lot because it began to bring to light the whole issue of problems that can occur when having sex or diseases or how to remove the taboo a little bit” (Maria).

However, when asked if they knew about toxic relationships, they only remembered Adam and Eric’s relationship. According to them, the character could generate this aggressiveness because he could have problems recognising himself as homosexual because he had a traditional father or because society has made him aggressive. At the beginning of the interview, one of the girls interviewed pointed out that society is responsible for this aggressiveness for not letting him be the way he is, homosexual. “I think he is aggressive because of what society has done to Adam, for not letting him be the way he is” (Maria).

The young people have become more aware of toxic relationships and their effects on health after learning about the scientific information provided during the interview, especially when identifying Adam’s DTM, the health effects of aggressive masculinity, the socialisation capacity of the series in young people, and the existence of this type of relationship in their environment.

After watching the two videos of the two affective-sexual relationships analysed, all four young people detected Adam’s DTM, mainly because of his aggressiveness against the boys with whom Aimee and Eric have relationships. One of the boys interviewed identifies the character’s DTM and further adds that the aggressiveness is Adam’s own fault for being jealous. “Adam is a traditional dominant masculinity. Neither Aimee nor Eric is to blame, it is Adam’s own fault because he is jealous” (Jon). All four interviewees identified this type of masculinity in actual cases that they know of in real relationships in their environments, be those of family, friends, or acquaintances. One of the girls mentioned how she knows of close relationships which are toxic in which the partner is controlled or ordered not to talk to the opposite sex. “Yes, I have heard in relatives of mine where they tell their partners that as long as we are together, don’t talk to the opposite sex” (Nancy).

The young people interviewed identified the health consequences of being in a relationship with Adam. One of the boys pointed out health problems seen in the case of Aimee and Eric and pointed out that they were getting worse and worse. “Eric’s health is getting worse and worse. You can also see it in the other girl” (Jon). This identification allowed them to analyse the relationships they know in real life. Remarkably, one of the boys interviewed explained how he has friends who have told him that having relationships like this makes them tired, disconcerted, and sick. “I have had people who over time it has affected them physically. They are more tired, or they don’t know what to do and of course, in the end, it affects you everywhere. There are people who get sick”. (Abraham).

At the same time, the young people interviewed talked about how watching these series can further socialise violence in relationships, whether heterosexual or homosexual. One of the girls interviewed pointed out that these chapters of violence are given in the series as something normal, and the audience doesn’t realise it. “It is presented as something very normal, very good, nothing happens, but if you analyse it a little, it is very hard” (Maria). That is why they pointed out that audiovisual professionals should make these relationships not normal and not present them as “cool”. One of the boys pointed out that audiovisual productions should not add attractiveness or show normalisation of toxic relationships. “To begin with, the series should not show that these relationships are cool or normal, especially because not all of them are like that” (Abraham).

These dialogical interactions with scientists and young people have helped them to more easily identify violent relationships and their effects on health. Therefore, they point out that it is important to talk about these issues, but on a scientific basis. One of the interviewees mentioned that these issues should be discussed with professionals or have a scientific basis. “I think these issues should be discussed with a professional or scientist” (Jon).

## 4. Discussion

The evidence-based dialogical interactions with the young people interviewed have contributed to reflection and dialogue about toxic relationships and their effects on health. This dialogue based on scientific evidence has provided tools to analyse and identify this problem that appears both in the series and in real life. This corroborates that dialogue about healthy and toxic relationships can promote change and improvement in young people towards healthy relationships [35,54].

Science-based dialogues allow for the expansion of knowledge that they did not previously have about toxic relationships and their effects on health, which is not accomplished through other types of interactions, so bringing science to citizens can provide greater critical capacity and media literacy because it is not just about having interactions, but about having quality interactions based on science.

Serials have an impact on the socialisation of young people [15], especially on sexual behaviours [16]. *Sex Education*, while providing information on sexual practice or technique [23], does not discuss with whom to have healthy and violent-free relationships nor does it discuss or condemn Adam’s aggressive behaviour, which can socialise young people into gender-based violence. So although the series wants to break sexual taboos, it continues to reproduce the same behaviours, attraction, and violence in affective-sexual relationships as other series [26,27,28].

Young people who interact with scientists on the subject of contact with science in sexual and emotional relationships and health issues can provide young people with a tool that allows them to analyse and identify DTM [34,40] and so allows them to differentiate unhealthy relationships from healthy ones. This is an identification that, in addition to applying to relationships in fiction, can also be applied in real life, as the young people interviewed in this research have.

Therefore, fostering science-based dialogical interactions among young people and the communicative analysis of audiovisual products favours the critical capacity of boys and girls regarding the affective-sexual relationships they have and those of people around them. Talking about and seeing through series such as *Sex Education* the physical and mental health problems generated by toxic relationships provokes rejection in all young people. Therefore, the promotion of spaces between science and young people can favour the prevention of illnesses, as well as gender violence.

This research advances the knowledge between affective-sexual relationships and health in audiovisual products. At the same time, it helps young people to transform their relationships and those around them. However, it would be necessary to go further in future research with more young people and analyse the impact on socialisation in affective-sexual relationships of series that promote healthy relationships with NAM men and their effects on health.

## 5. Conclusions

Science-based dialogic interactions with young people about relationships and health facilitate the identification of DTM, toxic relationships, and their effects on health, n identification that can contribute to overcoming toxic relationships, and thus violence in relationships.

Therefore, it would be advisable for these dialogical interactions between young people and science on love and health to be promoted in those spaces where young people spend most of their time, such as educational centres. It is also observed that audiovisual productions such as fiction series should promote these spaces for reflection in order to provoke the audience’s thinking and critical capacity.

## Figures and Tables

**Table 1:** Category and subcategories of Communicative Content Analysis.

Category	Subcategory	Description
Consequence on Health	Mental Health	It refers to the mental health consequences of being in an affective-sexual relationship with a DTM.
	Physical Health	It refers to the physical health consequences of being in an affective-sexual relationship with a DTM.

**Table 2:** Profiles of the interviewees through the Communicative Interviews.

Anonymised Code (Interviewees)	Age
Nancy	19
Abraham	16
Jon	20
Maria	21

**Table 3:** Categories of Communicative Interviews.

Categories	Description
Exclusion	The verbal language does not relate DTM to negative health consequences.
Transformation	The verbal language does relate the relationship displaying DTM with negative health consequences or healthy relationships with positive health consequences.

## Data Availability

The data presented in this article are available under request to the corresponding author. However, the data are not publicly available due to ethical requirements of privacy and protection of the anonymity of the participants.
